# Predictive Value of Frailty, Comorbidity, and Patient-Reported Measures for Hospitalization or Death in Older Outpatients: Quality of Life and Depression as Prognostic Red Flags

**DOI:** 10.3390/diagnostics15151857

**Published:** 2025-07-23

**Authors:** Dimitrios Anagnostou, Nikolaos Theodorakis, Sofia Kalantzi, Aikaterini Spyridaki, Christos Chitas, Vassilis Milionis, Zoi Kollia, Michalitsa Christodoulou, Ioanna Nella, Aggeliki Spathara, Efi Gourzoulidou, Sofia Athinaiou, Gesthimani Triantafylli, Georgia Vamvakou, Maria Nikolaou

**Affiliations:** 1 Geriatric Outpatient Clinic 65+, Sismanogleio-Amalia Fleming General Hospital, 14 25is Martiou Str., 15127 Melissia, Greece; jimdimitris100@gmail.com (D.A.); nikolaostheodorakis1997@yahoo.com (N.T.);; 2 Department of Cardiology, Sismanogleio-Amalia Fleming General Hospital, 14 25is Martiou Str., 15127 Melissia, Greece; 3 School of Medicine, National and Kapodistrian University of Athens, 75 Mikras Asias, 11527 Athens, Greece; 4 Department of Internal Medicine, Sismanogleio-Amalia Fleming General Hospital, 14 25is Martiou Str., 15127 Melissia, Greece; 5 Department of Psychiatry, Sismanogleio-Amalia Fleming General Hospital, 1 Sismanogleiou Str., 15126 Marousi, Greece

**Keywords:** frailty, patient-reported outcomes (PROs), quality of life, depressive symptoms, geriatric assessment, hospitalization, mortality, older adults, outpatient care, predictive modeling

## Abstract

**Objectives**: To identify clinical, functional, laboratory, and patient-reported parameters associated with medium-term risk of hospitalization or death among older adults attending a multidisciplinary outpatient clinic, and to assess the predictive performance of these measures for individual risk stratification. **Methods**: In this cohort study, 350 adults aged ≥65 years were assessed at baseline and followed for an average of 8 months. The primary outcome was a composite of hospitalization or all-cause mortality. Parameters assessed included frailty and comorbidity measures, functional parameters, such as gait speed and grip strength, laboratory biomarkers, and patient-reported measures, such as quality of life (QoL, assessed on a Likert scale) and the presence of depressive symptoms. Predictive performance was evaluated using univariable logistic regression and multivariable modeling. Discriminative ability was assessed via area under the ROC curve (AUC), and selected models were internally validated using repeated k-fold cross-validation. **Results**: Overall, 40 participants (11.4%) experienced hospitalization or death. Traditional clinical risk indicators, including frailty and comorbidity scores, were significantly associated with the outcome. Patient-reported QoL (AUC = 0.74) and Geriatric Depression Scale (GDS) scores (AUC = 0.67) demonstrated useful overall discriminatory ability, with high specificities at optimal cut-offs, suggesting they could act as “red flags” for adverse outcomes. However, the limited sensitivities of individual predictors underscore the need for more comprehensive screening instruments with improved ability to identify at-risk individuals earlier. A multivariable model that incorporated several predictors did not outperform QoL alone (AUC = 0.79), with cross-validation confirming comparable discriminative performance. **Conclusions**: Patient-reported measures—particularly quality of life and depressive symptoms—are valuable predictors of hospitalization or death and may enhance traditional frailty and comorbidity assessments in outpatient geriatric care. Future work should focus on developing or integrating screening tools with greater sensitivity to optimize early risk detection and guide preventive interventions.

## 1. Introduction

As global populations continue to age [1], the clinical management of older adults is becoming increasingly complex. This demographic frequently presents with multimorbidity, reduced physiological reserve, and increased susceptibility to adverse health outcomes [2]. Accurately predicting these outcomes—such as hospitalization or death—remains a central challenge in geriatric medicine and risk stratification. Traditional comorbidity scores, such as the Charlson Comorbidity Index (CCI), have been shown to predict long-term survival but they are not specific to older adults [3]. Frailty, characterized by decreased resilience to stressors [4], has emerged as a key predictor of adverse clinical trajectories in this population [5,6]. Indeed, frailty assessment tools, such as the Clinical Frailty Scale (CFS) and Fried frailty phenotype, have been validated in older cohorts [7,8]. In addition, laboratory, cognitive, and functional parameters also hold prognostic value, as can be seen in models like the Essential Frailty Toolset (EFT), which combines hemoglobin, albumin, chair rise time, and Mini-Mental Status Examination (MMSE) score to predict one-year mortality in older adults undergoing aortic valve replacement [9].

Importantly, subjective—not strictly quantifiable—measures may significantly aid prognostication. For instance, the Clinical Frailty Scale (CFS) relies primarily on the physician’s clinical judgment rather than strict numerical cut-offs [7]. Similarly, patient-reported components, such as self-rated exhaustion and physical activity, are central to the Fried frailty phenotype, suggesting that frailty includes subjective dimensions not fully captured by conventional functional or laboratory assessments. In fact, patients themselves may offer valuable insights into their own health status, and incorporating their perspectives could facilitate earlier identification of risk [10]. Although the association between patient-reported measures and adverse events has been examined in previous studies [11,12], fewer studies have incorporated these measures into prognostic models that also include frailty, comorbidity, and biological markers within a single analytical framework. This study contributes to the literature by jointly assessing clinical, biological, functional, and patient-reported measures in older outpatients and comparing their predictive value using multivariable regression techniques. By integrating what the physician observes, what laboratory and functional assessments reveal, and what the patient personally reports, this approach triangulates risk from multiple dimensions. This is particularly valuable in individuals who may appear clinically well—those without evident frailty or significant comorbidity—yet remain vulnerable to adverse outcomes. Such integration allows for earlier and more accurate identification of at-risk patients, ultimately supporting a more comprehensive and person-centered model of care.

## 2. Objectives

The objective of this study was to identify clinical, functional, laboratory, and self-reported factors associated with medium-term adverse outcomes—specifically, hospitalization or death—in older adults. Particular emphasis was placed on the contribution of patients’ own perspectives through assessment of self-reported quality of life and the existence of depressive symptoms. We hypothesized that incorporating more of the patient’s perspective may enhance the identification of high-risk patients for such events.

## 3. Materials and Methods

This cohort study was conducted at the multidisciplinary 65+ Clinic of Amalia Fleming General Hospital in Athens, Greece [13]. The study included 388 patients aged 65 years or older who attended the outpatient clinic between March 2023 and August 2024. The final sample consisted of 350 patients, after excluding 3 individuals with missing basic demographic data and 35 who were lost to follow-up. Each patient underwent a baseline evaluation and a follow-up assessment, both conducted by a multidisciplinary team comprising an internist, cardiologist, psychiatrist, psychologist, physiotherapist, and specialized nurses. Events were ascertained through direct patient interviews, through review of hospital records, and telephone contact with the patients or caregivers. Participants were followed for an average of 8 months. No standardized fixed-point assessment was scheduled, and hence the timing of outcome assessment varied between individuals. Ethical approval was obtained from the local institutional review board, and the study was conducted in accordance with the principles of the Declaration of Helsinki.

### 3.1. Primary Outcome

The primary outcome of interest was a composite binary endpoint of all-cause mortality or hospitalization between the baseline evaluation and the second assessment. We combined these outcomes to capture a broader picture of clinically meaningful health deterioration in older adults, enhance statistical power by increasing the overall event rate, and account for the competing risk of death precluding subsequent hospitalization (Supplementary Materials).

### 3.2. Study Variables

Study variables included age and years of education, both analyzed as continuous variables, and gender, analyzed as a categorical variable. Family and social life were also assessed and treated as categorical variables. Frailty was assessed using the Fried frailty scale, the Clinical Frailty Scale (CFS), and the Essential Frailty Toolset (EFT), each treated as a continuous (point-based) variable. Comorbidity burden was assessed using the Charlson Comorbidity Index (CCI), also treated as a continuous (point-based) variable. Objective measures of functional status included gait speed (measured in meters per second, derived from the time taken to walk a fixed 400-m distance) and grip strength (measured in kilograms using a dynamometer). These measures were also considered as low or normal and treated as binary variables in accordance with the criteria defined in the Fried scale [4]; gait speed cut-offs varied depending on body mass index (BMI) and height, while grip strength thresholds differed based on BMI and gender. Additionally, a plethora of laboratory parameters were recorded including hemoglobin (mg/dL), hematocrit (%), high sensitivity troponin T (ng/L), serum albumin (g/dL), ferritin (mcg/L), vitamin D levels (ng/mL) and estimated glomerular filtration rate (eGFR) (mL/m^2^/1.73 m^2^) using the CKD-EPI equation; all were treated as continuous variables. Estimated glomerular filtration rate (eGFR) values below 60 mL/min/1.73 m^2^ were also classified as decreased and treated as a binary variable. C-reactive protein (CRP) was treated as a binary variable, with levels above 3 mg/dL considered detectable. Lastly, the Geriatric Depression Scale (GDS) was administered and analyzed both as a continuous measure and as a dichotomous variable, with scores of 15 or higher indicating depression [14]. Quality of life was assessed through a self-reported 10-point Likert scale, by directly asking participants “How would you rate your overall quality of life?”, with higher scores indicating better quality of life. The scale was treated as a continuous variable in this analysis. No specific cut-offs were used and the measure served as a subjective global rating of perceived quality of life.

## 4. Statistical Analysis

Baseline characteristics, descriptive statistics, and univariable (unadjusted) logistic regression analyses were conducted using STATA version 13 to explore associations between individual predictors and the outcome of interest. A single final multivariable (adjusted) logistic regression model was then constructed in R version 4.3 using the glmulti package (version 1.08). In accordance with the rule of approximately 10 events per variable (EPV), and given the total of 40 observed events, the number of predictors was restricted to four to avoid overfitting [15]. Among all possible models satisfying this criterion, the best-fitting model was selected based on the Akaike Information Criterion (AIC). Model fit was assessed using the likelihood ratio χ^2^ test for overall performance and the Hosmer–Lemeshow χ^2^ test for goodness of fit. Linearity assumption for continuous predictors in the multivariable model was examined using the Box–Tidwell test. Missing data were handled via case-wise deletion (complete case analysis). A significance level of *p* ≤ 0.05 was applied to all statistical analyses. As part of the sensitivity analysis, potential outliers and influential observations were examined. Outliers were identified based on deviance residuals, while influence was assessed using leverage (hat) values. To assess multicollinearity among independent variables, variance inflation factors (VIFs) were calculated. Model discriminatory ability was evaluated using the area under the receiver operating characteristic curve (AUC). To assess internal validity and ensure robustness of model performance, repeated k-fold cross-validation was performed on selected candidate models using the caret package (version 7.0.1) in R. Complementary AUC estimates based on pooled out-of-fold predictions were reported.

## 5. Results

The mean follow-up duration of participants was 8 months. Overall, 40 out of 350 patients (11.4%) experienced death or hospitalization within the study period, comprising 9 deaths and 31 hospitalizations. Baseline characteristics of the study population are presented in Table 1.

The results of the univariable (unadjusted) analyses are presented in Table 2. No correction for multiple comparisons was applied. Each additional year of age was associated with a 6% increase in the odds of experiencing hospitalization or death within one year (OR 1.06). None of the remaining demographic variables demonstrated a statistically significant association with the outcome. In contrast, all physician-assessed frailty and comorbidity scores—including the Fried score, Clinical Frailty Scale (CFS), Essential Frailty Toolset (EFT), and Charlson Comorbidity Index—were significantly associated with the outcome (ORs 1.68–1.73), indicating that each one-point increase in these scores corresponded to a 68% to 73% increase in the odds of hospitalization or death. Among objective measures, only gait speed and weight loss reached statistical significance. Each 1 m/s increase in gait speed was associated with an 85% reduction in the odds of the outcome (OR 0.15), whereas gait speed below the Fried scale threshold was associated with nearly fourfold higher odds (OR 3.71). Similarly, unintentional weight loss ≥ 5 kg (or a BMI < 18.5 kg/m^2^) was associated with nearly three times higher odds of the outcome (OR 2.80). Additionally, several laboratory parameters were associated with the outcome. Each 1 g/dL increase in albumin was associated with a 78% reduction in odds (OR 0.22), while each unit decrease in eGFR corresponded to a 3% increase in odds (OR 0.97). Participants with eGFR < 60 mL/min/1.73 m^2^ had nearly fivefold higher odds of hospitalization or death (OR 4.89). Detectable CRP levels were similarly associated with more than fourfold higher odds (OR 4.29). Not surprisingly, prior hospitalizations were also strong predictors of the outcome. Any hospitalization in the past year was associated with more than a threefold increase in odds (OR 3.26), and hospitalizations due to cardiovascular causes showed a comparable association (OR 2.97). Finally, measures reflecting the patient’s perspective were significant as well. Each point increase in the Geriatric Depression Scale (GDS) score was associated with an 11% increase in odds (OR 1.11), while depression (GDS ≥ 15) was associated with more than threefold higher odds of the outcome (OR 3.45). Lastly, each one-point decrease in self-rated quality of life corresponded to a 70% increase in odds (OR 1.70).

Comparing the discriminative ability of these variables is not straightforward, as they are measured on different scales. To address this, area under the curve (AUC) values were calculated for each significant predictor and are presented in Table 3. Technically, the AUC reflects the probability that a randomly selected patient who experienced the outcome will have a higher value on the given predictor than a randomly selected patient who did not. Practically, an AUC of 0.5 indicates no discriminative ability (equivalent to chance), while values closer to 1.0 indicate stronger discrimination. In this univariable analysis the Charlson Comorbidity Score (AUC 0.7) and self-reported quality of life (QoL) (0.74) demonstrated the highest AUCs among all predictors.

In the multivariable analysis, a final predictive model was constructed. All predictors that were statistically significant in the univariable analyses were combined in all possible combinations, and the best model was selected based on the Akaike Information Criterion (AIC). To limit the risk of overfitting, the number of predictors was capped at four, appropriate for the number of observed events (40). Results are presented in Table 4.

### 5.1. Multivariable Model Checking and Sensitivity Analysis

The overall model likelihood ratio test was statistically significant (*p* < 0.001), indicating that the predictors were jointly associated with the outcome. Model calibration was adequate, as assessed by the Hosmer–Lemeshow goodness-of-fit test using 10 groups, with no evidence of poor fit (*p* = 0.63). Linearity of log odds for continuous predictors was examined using the Box–Tidwell test. No significant deviations from linearity were detected overall; however, the interaction term GDS × log(GDS) yielded a *p*-value of approximately 0.07, suggesting a potential non-linear relationship that may merit further consideration. After examining component-plus-residual plots, the linearity assumption for GDS was deemed acceptable. Deviance residuals were examined to assess model fit and identify potential outliers. A total of 10 observations were found to have absolute deviance residuals greater than 2. Upon further inspection, these observations did not exhibit any data entry errors or implausible values and were thus retained in the analysis. Leverage values (hat values) were assessed to identify observations with potentially high influence based on predictor values. Using the threshold of approximately 3*p/n, where p is the number of predictors including the intercept and n is the sample size, 21 observations were identified as having high leverage. The analysis was rerun after excluding these observations, with no significant differences observed in the model estimates. As such, all observations were retained in the final analysis. Finally, variance inflation factors (VIFs) were computed for all predictors, with values approximately equal to 1, indicating no evidence of multicollinearity.

### 5.2. Internal Validation

To evaluate the discriminatory performance of candidate prognostic models, we conducted a repeated k-fold cross-validation procedure with 5 folds and 100 repetitions. In each repetition, the dataset was randomly partitioned into five mutually exclusive subsets (folds) of approximately equal size. Each model was trained on four folds and tested on the remaining fold, cycling through all five folds. This process was repeated 100 times with different random splits to reduce variability and increase the stability of performance estimates. For each model, we obtained out-of-fold predicted probabilities for every observation by aggregating predictions from all test folds across all repetitions. These predictions were then compared to observed outcomes to assess model discrimination using the area under the receiver operating characteristic (ROC) curve (AUC), along with corresponding 95% confidence intervals (CIs) based on the pooled results.

Three logistic regression models were evaluated:

1. A univariable model including only the Likert quality of life (QoL) score,
2. A univariable model including only the Charlson Comorbidity Index, and
3. A multivariable model incorporating Geriatric Depression Scale (GDS) score, CRP status (binary), gait speed (m/s), and albumin level (g/dL).

**Figure 1:**
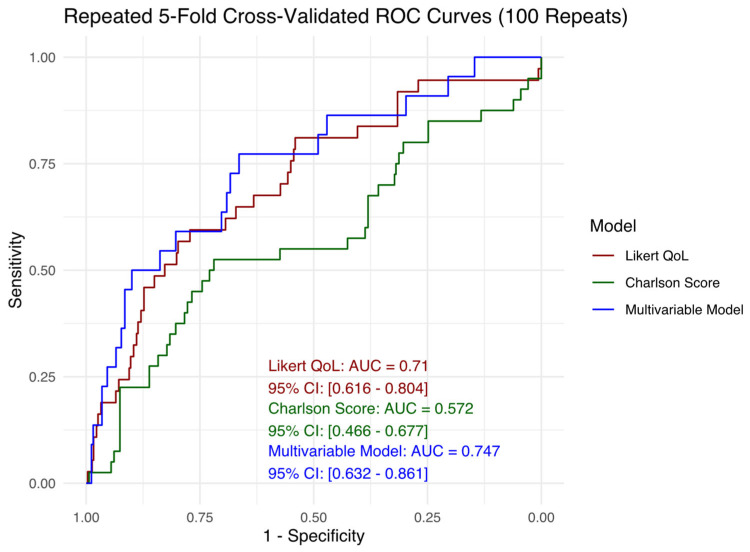
Repeated 5-fold cross-validated ROC curves for three logistic regression models predicting the 1-year event, using 100 repetitions of 5-fold cross-validation. The red curve represents a univariable model using the Likert quality of life (QoL) score (AUC = 0.710, 95% CI: 0.616–0.804). The green curve corresponds to a univariable model based on the Charlson Comorbidity Index (AUC = 0.572, 95% CI: 0.466–0.677). The blue curve represents a multivariable logistic regression model including GDS, CRP status, gait speed, and albumin (AUC = 0.747, 95% CI: 0.632–0.861). Curves reflect model performance based on out-of-fold predictions pooled across repetitions. The area under the curve (AUC) quantifies each model’s ability to discriminate between individuals who did and did not experience the event within 1 year.

Model 1 (Likert QoL score) and Model 2 (Charlson Comorbidity Index) were selected because they demonstrated the highest discriminatory performance (i.e., highest AUC values) among all candidate predictors in univariable analyses. Model 3 was explored to assess whether incorporating multiple predictors could meaningfully improve discriminatory performance. As shown in Figure 1, ROC curves were generated for each model based on the pooled predictions. The Charlson Comorbidity Index yielded the lowest AUC, indicating the lowest discriminatory ability in this context. In contrast, the Likert QoL score achieved performance comparable to the multivariable model. The full ROC curves, AUC estimates, and 95% CIs are displayed in Figure 1.

## 6. Discussion

In this study of older outpatients, we estimate that approximately 11.4% of individuals will experience hospitalization or death within an 8-month period (95% CI: 8% to 15%, Clopper–Pearson method). This finding highlights a clinically meaningful risk, even in a population with relatively low levels of frailty and comorbidity at baseline (median Charlson Comorbidity Index [CCI] = 3, median Clinical Frailty Scale [CFS] = 2).

Identifying and addressing potentially modifiable risk factors in this group may offer an opportunity for early intervention and improved outcomes. While the observational nature of this study precludes causal inference, the consistent associations observed between adverse outcomes and measures of frailty, comorbidity burden, depressive symptoms, and perceived quality of life suggest that these domains warrant further investigation. Notably, both the Charlson score and all frailty measures remained significant predictors, underscoring their prognostic value even among relatively healthy older adults. Whether targeted interventions aimed at improving frailty status and managing comorbidities can effectively prevent adverse outcomes remains to be established. However, evidence suggests that multicomponent interventions—including resistance exercise, nutritional support, medication optimization, and psychosocial engagement—can improve frailty markers and functional outcomes, particularly in pre-frail or mildly frail individuals, though results on hard outcomes like mortality or hospitalization remain mixed [16,17].

Depressive symptoms have consistently been associated with increased risks of hospitalization and mortality [18,19], and similar associations have been published for self-reported quality of life measures [20]. Again, it is difficult to establish causality in such associations from observational studies. For instance, depressive symptoms may exacerbate physical inactivity, poor nutrition, and social withdrawal, and thereby accelerate frailty and increase vulnerability to adverse outcomes. On the other hand, depression may also reflect an underlying burden of disease, serving as a mediator between underlying frailty and adverse events. Similar arguments can be made for quality of life measures. However, we consider it more plausible that a patient’s perceived quality of life functions as a composite signal, reflecting the cumulative burden of diverse physical, psychological, and social deficits. Nevertheless, we acknowledge that quality of life may also play an independent role in mediating the risk of adverse outcomes. At a minimum, efforts to identify the most influential contributors to this subjective measure—such as pain, functional limitation, or emotional distress—may provide valuable targets for intervention. Use of digital health tools, such as “smart-aging” platforms, could enhance our ability to detect such deficits [21,22]. Randomized controlled trials are needed to determine whether targeting these domains can effectively reduce the risk of adverse outcomes.

In terms of predictive performance, all evaluated parameters demonstrated, at best, modest sensitivities at their respective optimal thresholds (Table 3). This highlights a notable gap in screening individuals at risk. The most sensitive variables were age (sensitivity ~70%), followed by self-reported quality of life and the Clinical Frailty Scale (CFS) score (each with sensitivity ~60%). While these measures show relative promise, their limited sensitivity underscores the need for more comprehensive or combined approaches to improve early risk detection in outpatient settings. Interestingly, the data-driven optimal cutoff for age, closely aligns with the conceptual distinction between “young-old” and “old-old” adults frequently proposed in geriatric research [23,24].

Encouragingly, several measures demonstrated good specificity values. Both comorbidity and frailty scores showed at least moderate specificities (~64–77%). These tools may be particularly useful for ruling in individuals at elevated risk, and it is reasonable to hypothesize that their predictive performance could be even greater in sicker, more frail cohorts. The value of objective physical measures—such as low gait speed and unintentional weight loss—also lies primarily in their ability to identify individuals at increased risk. These indicators demonstrated very high specificities (~90%), suggesting that when such deficits are present, the likelihood of adverse outcomes is substantially increased. This pattern likely reflects their emergence in more advanced stages of physiological decline, making them strong “red flags” in clinical assessment [25,26]. For laboratory markers, moderate-to high-specificity was observed for markers of inflammation (detectable CRP; specificity 77.9%), nutritional or inflammatory status (low albumin; specificity 72.2%), and renal dysfunction (reduced eGFR; specificity 86.4%). These biomarkers are well-established risk factors for adverse outcomes in older adults [27,28]. Notably, inflammaging—defined as chronic, low-grade systemic inflammation that develops with age—is increasingly recognized as a key contributor to frailty and age-related vulnerability [29,30]. Finally, patient self-reported measures—namely quality of life and depressive symptoms—demonstrated moderate-to-high specificities, with values of approximately 77% for quality of life and 81% for the Geriatric Depression Scale (GDS). The optimal cut-off for GDS in our cohort was 11.5 points, suggesting that even moderate levels of depressive symptoms carry prognostic value. Moreover, the even higher specificity observed with a GDS score greater than 15 (87.8%) further highlights the potential of more severe depressive symptoms acting as a “red flag” in identifying individuals at elevated risk. The predictive value of self-reported health for mortality and other adverse outcomes has been consistently replicated in multiple longitudinal studies and meta-analyses across diverse populations [31,32,33,34,35]. These findings support the integration of psychological well-being and self-reported quality of life measures into routine geriatric assessments, alongside physical and biological markers, to provide a more holistic and effective approach to risk stratification in older adults [36].

**Figure 2:**
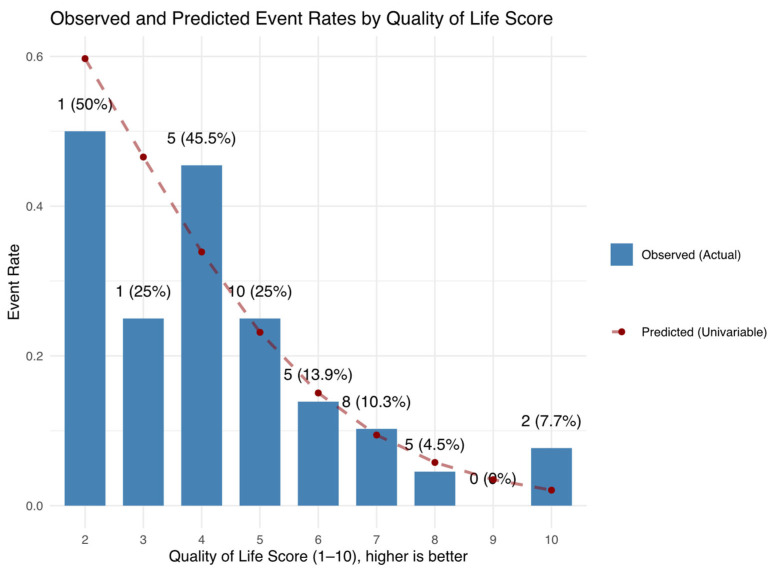
Observed event rates (based on actual data) and predicted probabilities (from a univariable logistic regression model using Likert QoL as the sole predictor) for death or hospitalization. This parsimonious model demonstrated a repeated cross-validated AUC of 0.71. At optimal cutoff (6.45) sensitivity was 59.5% and specificity was 76.9%. **Abbreviations**: QoL = quality of life (self-reported on a 10-point scale).

In the final multivariable model, which was constructed using a systematic statistical approach (minimizing the AIC and limited to four predictors), representatives of many domains remained: CRP, GDS, gait speed, and albumin. The model’s discriminative ability, however, (AUC = 0.79; 95% CI: 0.69–0.90) was not significantly different from that of self-reported quality of life alone (AUC = 0.74; 95% CI: 0.65–0.83). This observation remained consistent after internal model validation. Hence, a simple measure like the Likert QoL (Figure 2) may capture much of the prognostic information provided by more complex models, supporting its potential value as a parsimonious predictor, at least in outpatient adults. It is also possible that our sample was not large enough to detect a statistically significant difference between these models. Conversely, the multivariable model seemed to outperform the Charlson comorbidity score. The relatively good health status of the cohort (median Charlson score = 3) might have contributed to this. Whether similar results would be observed in sicker populations remains an open question. In either case, the multivariable model may serve as a starting point for the development of a predictive score in these patients, one which includes patient-reported measures. The advantage of such models lies in their balanced sensitivity and specificity profile (in this case, sensitivity is ~68%; specificity is ~80%).

The need to move beyond traditional frailty scales and to develop predictive tools tailored to specific patient populations has been emphasized in previous studies [37]. In the context of community-dwelling outpatients, self-reported measures may gain particular relevance in the absence of more specific or objective clinical markers. Based on our findings, patients over the age of 74 or those with a Clinical Frailty Scale (CFS) score of 2 or more should undergo further assessment, during which quality of life and depressive symptoms are specifically evaluated. Individuals reporting a quality of life score below 6 (in a 10-point scale) or a Geriatric Depression Scale (GDS) score of 12 or more should be considered at high risk for future adverse events. In such cases, appropriate preventive interventions should be initiated, including ensuring timely access to healthcare and psychosocial support.

## 7. Limitations

This study has several limitations that should be acknowledged. First, as an observational study, it is inherently limited in its ability to establish causal relationships between predictors and outcomes. Second, the study population consisted of older adults who were already engaged with or referred to a geriatric outpatient clinic, which may introduce selection bias, as these individuals could differ systematically from older adults in the general population. Additionally, the study was conducted at a single outpatient center, which may limit the generalizability of the findings to other settings or healthcare systems. Third, missing data were handled using complete case analysis, resulting in a reduced sample size for the final multivariable model (282 out of 350 patients). No imputation or sensitivity analysis was performed for the 35 patients that were lost to follow-up. Moreover, the number of observations included in candidate multivariable models varied slightly throughout the model selection process, depending on data availability for each predictor. Fourth, the number of candidate predictors was limited to four to reduce the risk of overfitting, based on the number of observed events. If more events had been available, it would have been possible to explore a larger set of predictors or potential interactions. Fifth, some potentially relevant predictors may have been missing or unmeasured in the dataset, limiting the comprehensiveness of the model. Sixth, the self-reported nature of quality of life introduces the possibility of subjective variability, which may be influenced by social, cultural, or psychological factors not accounted for in the analysis. Although all participants received follow-up within the study period (March 2023 to August 2024), no standardized, fixed-point assessments were conducted, resulting in variability in the timing of outcome ascertainment across individuals. While this design provides valuable medium-term prognostic information, longer-term follow-up or the application of time-to-event methods (e.g., survival analysis) could offer a more nuanced and comprehensive understanding of risk trajectories over time. Finally, although internal validation was performed using repeated cross-validation, external validation in independent cohorts is needed to confirm the generalizability and robustness of the results.

## 8. Conclusions

**Figure 3:**
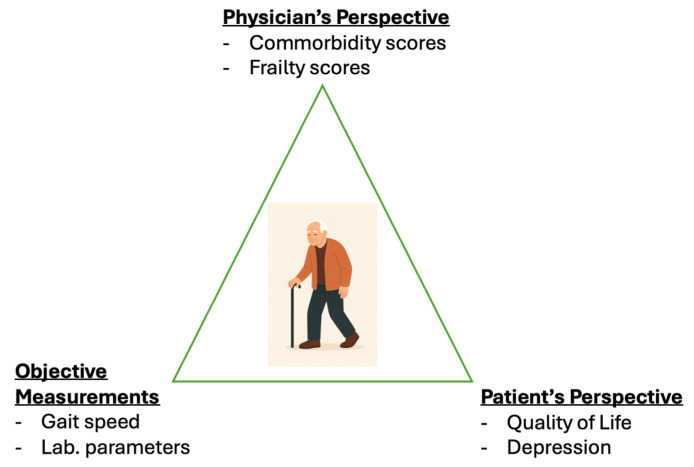
Components of a comprehensive geriatric assessment, including the physician’s perspective (e.g., comorbidity and frailty scores), objective measurements (e.g., gait speed and laboratory parameters), and the patient’s perspective (e.g., quality of life and depressive symptoms). While the first two components are traditionally emphasized in clinical practice, the patient’s perspective is equally important—particularly in the outpatient setting. Routine screening for depression and quality of life may provide valuable insights into overall health and support more person-centered care.

In conclusion, the patient’s perspective should be an integral component of geriatric assessment (Figure 3). A patient’s overall quality of life may serve as an aggregate indicator, reflecting the cumulative burden of multiple underlying deficits. Diminished quality of life and presence of depressive symptoms are “red flags” for future adverse outcomes. In addition, quality of life is among the few tools with at least moderate sensitivity for identifying high risk individuals, especially in an outpatient setting. There remains a clear need for better screening instruments. Multivariable predictive models that incorporate the patient’s perspective and psychological status may provide a foundation for predicting future adverse events. Randomized controlled trials could elucidate the effectiveness of targeted interventions.

## Figures and Tables

**Table 1:** Population Baseline Characteristics.

	Factor	No Event	Death/Hosp.	*p*-Value
	*N*	310	40	
Demographics	Age (years), mean (SD)	74.80 (6.88) (*n* = 310)	77.95 (8.75) (*n* = 40)	**0.01**
	Gender			0.56
	Male	139 (44.8%)	16 (40.0%)	
	Female	171 (55.2%)	24 (60.0%)	
	Lives with			0.57
	Spouse	191 (63.7%)	26 (72.2%)	
	Children	27 (9.0%)	3 (8.3%)	
	Other	82 (27.3%)	7 (19.4%)	
	Social life			0.82
	Poor	48 (16.4%)	7 (20.0%)	
	Intermediate	54 (18.5%)	7 (20.0%)	
	Satisfactory	190 (65.1%)	21 (60.0%)	
	Family life			0.62
	Married	192 (62.1%)	24 (66.7%)	
	Non-married	12 (3.9%)	2 (5.6%)	
	Widowed	80 (25.9%)	6 (16.7%)	0.56
	Other	25 (8.1%)	4 (11.1%)	
	Years of education, median (IQR)	3.00 (2.00, 5.00) (*n* = 300)	3.00 (1.00, 5.00) (*n* = 36)	0.43
Physician Scores	FRIED frailty score [0–5], median (IQR)	1.00 (0.00, 1.00) (*n* = 304)	1.00 (1.00, 2.00) (*n* = 35)	**0.005**
	CFS score [0–9], median (IQR)	2.00 (2.00, 3.00) (*n* = 305)	3.00 (2.00, 4.00) (*n* = 37)	**<0.001**
	EFT score [0–5], median (IQR)	0.00 (0.00, 1.00) (*n* = 310)	1.00 (0.00, 1.00) (*n* = 40)	**0.011**
	Charlson score, median (IQR)	3.00 (3.00, 4.00) (*n* = 310)	4.00 (3.00, 4.00) (*n* = 40)	**0.003**
	MMSE [0–30], median (IQR)	28.00 (26.00, 29.00) (*n* = 303)	27.00 (26.00, 30.00) (*n* = 37)	0.28
Objective Metrics	Gait speed (m/s), mean (SD)	1.02 (0.24) (*n* = 304)	0.90 (0.30) (*n* = 30)	**0.01**
	Low gait speed (FRIED) *	39 (12.8%)	12 (35.3%)	**<0.001**
	Grip strength (kg), mean (SD)	22.35 (6.96) (*n* = 308)	20.49 (8.33) (*n* = 36)	0.14
	Low grip strength (FRIED) *	187 (61.1%)	26 (72.2%)	0.19
	Weight loss > 5 kg (FRIED) **	19 (6.3%)	6 (15.8%)	0.03
Laboratory	HCT (%), mean (SD)	41.73 (4.44) (*n* = 303)	40.49 (4.91) (*n* = 38)	0.11
	Hgb (mg/dL), mean (SD)	13.68 (1.53) (*n* = 303)	13.23 (1.70) (*n* = 38)	0.09
	hs tropT (ng/L), mean (SD)	14.83 (17.29) (*n* = 274)	22.65 (21.10) (*n* = 34)	**0.02**
	Albumin (g/dL), mean (SD)	4.17 (0.27) (*n* = 281)	4.06 (0.34) (*n* = 31)	**0.03**
	HbA1c (%), mean (SD)	5.83 (0.62) (*n* = 284)	5.89 (0.80) (*n* = 29)	0.65
	Ferritin (mcg/L), mean (SD)	114.87 (90.47) (*n* = 293)	106.85 (72.87) (*n* = 33)	0.62
	Vitamin D (ng/mL), mean (SD)	26.54 (10.07) (*n* = 276)	26.93 (15.89) (*n* = 27)	0.86
	eGFR (mL/m^2^/1.73 m^2^), mean (SD)	81.49 (17.77) (*n* = 308)	66.63 (26.45) (*n* = 38)	**<0.001**
	eGFR < 60	42 (13.6%)	17 (43.6%)	**<0.001**
	Detectable CRP+	64 (22.1%)	17 (54.8%)	**<0.001**
Past Events	Hosp. in the last year	36 (11.6%)	12 (30.0%)	**0.001**
	CV cause hosp. last year	24 (7.8%)	8 (20.0%)	**0.012**
Patient Perspective	GDS [0–30], median (IQR)	5.00 (2.00, 9.00) (*n* = 310)	11.00 (5.00, 15.50) (*n* = 40)	**<0.001**
	Depression (GDS 15–30)	38 (12.3%)	13 (32.5%)	**<0.001**
	Likert QoL [1–10], median (IQR)	8.00 (7.00, 8.00) (*n* = 307)	6.00 (5.00, 7.00) (*n* = 37)	**<0.001**

Continuous variables are reported as mean (standard deviation) if normally distributed, or as median (interquartile range) if non-normally distributed. Categorical variables are presented as numbers (percentages). For comparisons between groups, Pearson’s chi-squared test was used for categorical variables, two-sample *t*-test for normally distributed continuous variables, and Wilcoxon rank-sum test for non-normally distributed continuous variables. Abbreviations: SD = Standard deviation; IQR = Interquartile range; FRIED = Fried frailty phenotype; CFS = Clinical Frailty Scale; EFT = Essential Frailty Toolset; MMSE = Mini-Mental State Examination; HCT = Hematocrit; Hgb = Hemoglobin; hs tropT = High-sensitivity troponin T; HbA1c = Glycated hemoglobin; eGFR = Estimated glomerular filtration rate; CRP = C-reactive protein; CV = Cardiovascular; GDS = Geriatric Depression Scale; Hosp. = Hospitalization; QoL = Quality of life. *: low is defined in accordance with the criteria of the Fried scale; gait speed cut-offs vary depending on body mass index (BMI) and height, while grip strength thresholds differ based on BMI and gender. **: weight loss, as assessed in the Fried frailty scale, was defined as either self-reported unintentional weight loss of at least 5 kg (10 pounds) or a body mass index (BMI) < 18.5 kg/m^2^. +: detection threshold for CRP is 3.2 mg/L.

**Table 2:** Univariable Models (Outcome is death/hosp.).

	Factor	Odds Ratio	95% CI Lower	95% CI Upper	*p*-Value	*N*
Demographics	Age (years)	1.06	1.01	1.11	**0.01**	350
	Gender (Female)	1.22	0.62	2.39	0.56	350
	Lives with				0.55	336
	Children vs. spouse	0.82	0.23	2.88	0.75	
	Other vs. spouse	0.63	0.26	1.5	0.3	
	Social life				0.82	327
	Intermediate vs. poor	0.89	0.29	2.72	0.84	
	Satisfactory vs. poor	0.76	0.3	1.89	0.55	
	Family life				0.6	345
	Non-married vs. married	1.33	0.28	6.32	0.72	
	Widowed vs. married	0.6	0.24	1.52	0.28	
	Other vs. married	1.28	0.41	3.99	0.67	345
	Years of education	0.92	0.73	1.14	0.43	336
Physician Scores	FRIED score (0–5)	1.68	1.24	2.28	**<0.001**	339
	CFS score (0–9)	1.81	1.37	2.37	**<0.001**	342
	EFT score (0–5)	1.70	1.18	2.45	**0.005**	350
	Charlson score	1.73	1.23	2.44	**0.002**	350
	MMSE (0–30)	0.94	0.86	1.02	0.16	340
Objective Metrics	Gait speed (m/s)	0.15	0.03	0.64	**0.01**	334
	Low gait speed (FRIED) *	3.71	1.7	8.08	**<0.001**	338
	Grip strength (kg)	0.96	0.92	1.01	0.14	344
	Low grip strength (FRIED) *	1.65	0.77	3.55	0.20	342
	Weight loss > 5 kg (FRIED) **	2.80	1.04	7.53	**0.04**	341
Laboratory	HCT (%)	0.94	0.88	1.01	0.11	341
	Hgb (mg/dL)	0.84	0.69	1.03	0.1	341
	hs trop T (ng/L)	1.02	1	1.03	0.05	308
	Albumin (g/dL)	0.22	0.06	0.87	**0.03**	312
	HbA1c (%)	1.14	0.65	1.99	0.65	313
	Ferritin (mcg/L)	1	1	1	0.62	326
	Vitamin D (ng/mL)	1	0.97	1.04	0.86	303
	eGFR (mL/min/1.73 m^2^)	0.97	0.95	0.98	**<0.001**	346
	eGFR < 60	4.89	2.4	9.97	**<0.001**	347
	Detectable CRP+	4.29	2.01	9.17	**<0.001**	321
Past Events	Hosp. in last year	3.26	1.53	6.98	**<0.001**	350
	Hosp. last year for CV cause	2.97	1.23	7.16	**0.02**	349
Patient Perspective	GDS (0–30)	1.11	1.05	1.17	**<0.001**	350
	Depression (GDS 15–30)	3.45	1.64	7.25	**<0.001**	350
	Likert QoL (1–10) **per unit decrease**	1.7	1.37	2.11	**<0.001**	344

Univariable logistic regression models. *N* is number of participants included in each analysis. Differences in N across variables reflect missing data for some participants. Abbreviations: FRIED = Fried Frailty Phenotype; CFS = Clinical Frailty Scale; EFT = Edmonton Frailty Tool; MMSE = Mini-Mental State Examination; HCT = Hematocrit; Hgb = Hemoglobin; hs tropT = High-sensitivity Troponin T; HbA1c = Glycated Hemoglobin A1c; eGFR = Estimated Glomerular Filtration Rate; CRP = C-reactive Protein; Hosp. = Hospitalization; CV cause hosp. = Cardiovascular cause related hospitalization; GDS = Geriatric Depression Scale; QoL = quality of life. *: low is defined in accordance with the criteria of the Fried scale; gait speed cut-offs vary depending on body mass index (BMI) and height, while grip strength thresholds differ based on BMI and gender. **: weight loss, as assessed in the Fried frailty scale, was defined as either self-reported unintentional weight loss of at least 5 kg (10 pounds) or a body mass index (BMI) < 18.5 kg/m^2^. +: detection threshold for CRP is 3.2 mg/L.

**Table 3:** Discriminative Ability of Individual Predictors from Univariable Models.

	Factor	AUC	Optimal Cutoff	Sensitivity (%)	Specificity (%)
Demographics	Age	0.61 (0.51, 0.71)	73.5	72.5	52.3
Physician Scores	FRIED score (0–5)	0.64 (0.54, 0.74)	1.6	45.7	77.3
	CFS score (0–9)	0.66 (0.56, 0.76)	2.6	62.2	63.9
	EFT score (0–5)	0.60 (0.52, 0.69)	0.6	52.5	65.8
	Charlson score	0.70 (0.61, 0.78)	3.6	52.5	71.6
Objective Metrics	Gait speed (m/s)	0.62 (0.51, 0.73)	0.9	53.3	68.4
	Low gait speed (FRIED) *	0.61 (0.53, 0.70)	-	35.3	87.2
	Weight loss > 5 kg (FRIED) **	0.55 (0.49, 0.61)	-	15.8	93.7
Laboratory	Albumin (g/dL)	0.57 (0.45, 0.69)	4.0	48.4	72.2
	eGFR (mL/min/1.73 m^2^)	0.66 (0.55, 0.77)	68.9	55.3	78.2
	eGFR < 60	0.65 (0.57, 0.73)	-	43.6	86.4
	Detectable CRP+	0.66 (0.57, 0.76)	-	54.8	77.9
Past Events	Hosp. in last year	0.59 (0.52, 0.67)	-	30	88.4
	Hosp. last year for CV cause	0.56 (0.50, 0.63)	-	20	92.2
Patient Perspective	GDS (0–30)	0.67 (0.57, 0.76)	11.5	50.0	81.0
	Depression (GDS 15–30)	0.60 (0.53, 0.68)	-	32.5	87.8
	Likert QoL (1–10)	0.74 (0.65, 0.83)	6.45	59.5	76.9
Multivariable Model	[CRP, GDS, gait speed, albumin]	0.79 (0.69–0.90)	-	68.2	80.4

Area Under the Curve (AUC) reflects the discriminative ability of each predictor in univariable modeling; 95% confidence intervals are provided (DeLong’s method). For continuous predictors and for the final multivariable model, optimal cut-off values were determined using the Youden Index. Factors included in the final multivariable model are indicated in brackets [ ]. Abbreviations: FRIED = Fried Frailty Phenotype; CFS = Clinical Frailty Scale; EFT = Edmonton Frailty Tool; MMSE = Mini-Mental State Examination; HCT = Hematocrit; Hgb = Hemoglobin; hs tropT = High-sensitivity Troponin T; HbA1c = Glycated Hemoglobin A1c; eGFR = Estimated Glomerular Filtration Rate; CRP = C-reactive Protein; Hosp. = Hospitalization; CV cause hosp. = Cardiovascular cause related hospitalization; GDS = Geriatric Depression Scale; QoL = quality of life. *: low is defined in accordance with the criteria of the Fried scale; gait speed cut-offs vary depending on body mass index (BMI) and height. **: weight loss, as assessed in the Fried frailty scale, was defined as either self-reported unintentional weight loss of at least 5 kg (10 pounds) or a body mass index (BMI) < 18.5 kg/m^2^. +: detection threshold for CRP is 3.2 mg/L.

**Table 4:** Final multivariable model (Outcome is death/hosp.).

Factor	Odds Ratio	95% CI Lower	95% CI Upper	*p*-Value
Detectable CRP+	4.54	1.77	11.75	0.002
GDS (0–30)	1.13	1.05	1.23	0.002
Gait speed (m/s)	0.31	0.04	2.14	0.232
Albumin (g/dL)	0.80	0.13	4.76	0.807

Final model including the four predictors with the lowest Akaike Information Criterion (AIC). The model was fitted using maximum likelihood estimation in a sample of 281 participants. Adjusted odds ratios with 95% confidence intervals (CIs) are reported. Discriminative ability was assessed using the area under the receiver operating characteristic (ROC) curve (AUC = 0.79; 95% CI: 0.69–0.90). At the Youden threshold, sensitivity was 68.2% and specificity was 80.4%. +: detection threshold for CRP was 3.2 mg/L. Abbreviations: GDS = Geriatric Depression Scale; CRP = C-reactive Protein.

## Data Availability

Data are available from the corresponding author upon reasonable request, subject to institutional and privacy restrictions.

## Supplementary Materials

The following supporting information can be downloaded at: https://www.mdpi.com/article/10.3390/diagnostics15151857/s1. Included Hospitalization Causes (comma-separated). Excluded Hospitalization Causes (comma-separated).
