# Supplementary material for: Predictive Value of Frailty, Comorbidity, and Patient-Reported Measures for Hospitalization or Death in Older Outpatients: Quality of Life and Depression as Prognostic Red Flags
Source: Diagnostics (Basel). 2025 Jul 23;15(15):1857. doi: 10.3390/diagnostics15151857 (PMC12345878; doi:10.3390/diagnostics15151857)
Supplement: Supplementary file 1 [file diagnostics-15-01857-s001.zip › diagnostics-3728111-supplementary.pdf]

Included Hospitalization Causes (comma-separated)

Patients with one or more of the following reasons for hospitalization were included in the composite outcome:

Dyspnea, Pneumonia, Kidney cancer, Heart failure, Pulmonary hypertension, Respiratory infection, Acute myocardial infarction (MI), Allergy, Pacemaker implantation, Ovarian cancer, Lower limb edema, Orthostatic hypotension, Cardioversion for atrial fibrillation, Stroke, Breast cancer surgery, Pacemaker placement, Angioplasty, Defibrillator implantation, Ankle fracture, Breast cancer, Decompensated heart failure, Dyspnea with edema, Atrial fibrillation, Syncopal episode, Dilated cardiomyopathy, Fall, TAVI procedure, Post-surgical trauma infection, Renal colic, Lung cancer, Generalized cancer

Excluded Hospitalization Causes (comma-separated)

Patients with the following reasons for hospitalization were excluded from the composite outcome:

Cataract surgery, Knee arthroplasty, Blepharoplasty
